# Validation of an Analytical Method for Simultaneous Determination of 18 Persistent Organic Pollutants in Trout Using LLE Extraction and GC-MS/MS

**DOI:** 10.22037/ijpr.2019.1100748

**Published:** 2019

**Authors:** Mitra Bayat, Mohammad Saber tehrani, Farzad Kobarfard, Syed Waqif Husain, Hassan Yazdanpanah

**Affiliations:** a *Department of Chemistry, Islamic Azad University, Science and Research Branch, Tehran, Iran.*; b *Department of Medicinal Chemistry, School of Pharmacy, Shahid Beheshti University of Medical Sciences, Tehran, Iran. *; c *Phytochemistry Research Center, Shahid Beheshti University of Medical Sciences, Tehran, Iran. *; d *Food Safety Research Center (FSRC), Shahid Beheshti University of Medical Sciences, Tehran, Iran.*; e *Department of Toxicology and Pharmacology, School of Pharmacy, Shahid Beheshti University of Medical Sciences, Tehran, Iran.*

**Keywords:** Persistent Organic Pollutant (POPs), Polychlonitated biphenyls, Organochlorine pesticides, Trout, Liquid Liquid Extraction, GC-MS/MS, Iran

## Abstract

Persistent organic pollutants, (POPs), are vast distributed compounds in environment which are recognized as one of the global pollution problems. These groups of materials being dangerous due to their high stability are accumulated in animal tissues and occurring in the food chain. One of the major paths through which persistent organic combinations access to human body is consuming polluted foods, particularly, fishes. Among aquatic animals, trout as one of the mostly consumed fishes in Tehran’s food basket was studied. In this study, two categories of persistent organic pollutants: Organochlorine pesticides (OCPs) including HCB, Dieldrin, Methoxychlor, α-, ϒ-Chlordane, α-, β-Endosulfan and o,p’-DDE, p,p’-DDE, o,p’-DDT, p,p’-DDT and the second group Polychlonitated biphenyls (PCBs) including seven PCB congeners which are called indicator PCBs (IUPAC nos.: 28,52,101,118,138,153 and 180) were determined in trout by GC-MS/MS in MRM monitoring mode and LLE extraction. The average recoveries of OCPs and PCBs at five concentration levels (1, 2, 5, 10 and 20 ng/g for PCBs and 5 times for OCPs) were in the range of 73-112%. The relative standard deviations of POPs in fish were in the range of 1.4-17.9% for all of the concentration levels. The limit of detections (LODs) and the limit of quantitations (LOQs) were between 0.6-8.3 and 2-25 µg/kg, respectively. The results indicated the presence of organochlorine pesticides in trout and the levels of p,p’-DDE and p,p’-DDT were within the range of < LOQ -12.83 and < LOQ -10.2 ng/g ww (wet weight), respectively. According to the results, OCPs residues were lower than maximum residue levels set by European Council Directives.

## Introduction

Persistent organic pollutants are a group of hazardous organic compounds to human health and environments. In addition, POPs are typically semi-volatile, toxic, chemically and thermally stable with slow metabolic degradation, high lipophilicity and hydrophobicity (1). Due to these characteristics, they can be found in various environmental compartments like soil and sediments, atmosphere and water and they are also able to accumulate in the adipose tissues of biota (2). These chemicals are capable of long-range transport through the upper levels of atmosphere and can be condensed and also spread many kilometers away from source of contamination (3). The body of humans takes up POPs through respiration, skin absorption and ingestion of contaminated foods. The two first items are not the main transfer path and more than 90% of contaminations occur through contaminated food consumption (4). Seafoods (fish, shrimp, carb) are among the main sources of contaminations. Although these products account for only about 10% of the average human diet (5, 6), as it appears from the Bio-magnification pyramid (7), seafoods are among the most contaminated group of food stuffs when POPs are considered as their contaminants.

POPs could cause several harmful and adverse effects to human health and living organisms and they could be considered as carcinogenic, neurotoxic with harmful effects on reproductive and immunologic systems (8, 9).

Benzene ring, after Glycosyl group, is the second expanded chemical structure existing in the nature which constitutes 25% of biological earth mass. Lack of electrons’ instability in π orbitals of this ring increases its physical and chemical stability through resonance energy. Man utilized such stability to develop chemical materials for different purposes, not taking into consideration the outcome of this utilization: rapid increase in release of manmade aromatic components in ecosystems.

During the past four decades, persistent organic pollutants, have scattered and caused toxic effects on human beings and environment therefore they have raised many concerns with respect to public health. Despite limitations in the application of Organochlorines (OCs) from the beginning of 1970s in most of developed countries, currently, their application has been continued in agriculture causing health issues in most of the under development countries (10-13). In Stockholm conference, 13 groups of chemical materials were recognized as POPs: Polychlorinated dibenzo-p-dioxins (PCDDs), Polychlorinated dibenzo Furans (PCDFs), Polycyclic aromatic hydrocarbons (PAHs), Polybrominateddiphenyl ethers (PBDEs) or Brominated flame retardants (BFRs), Polychlorinated biphenyls (PCBs, and Organochlorine Pesticides (OCPs) include HCB, HCH, and its isomers and DDT and its metabolites, Cyclodienes insecticides containing Aldarin, Dieidrin, Endrin, Chlordane, Heptachlor, and the others Toxaphene and Mirex and many concerns have been raised about these compounds as environmental toxicant materials (14).

Within classical POPs, chlorinated compounds are the most relevant standing out OCPs and PCBs. Some of these components like PCBs are industrial contaminants and exert different variety of human activities. The first time they were synthesized in 1881 in Germany. Many commercial mixtures have been produced such as Aroclor, Clophen, Prodelec, Bayer, Pyralene, and others. Based on their biological activity and toxicity, PCB congeners can be divided into two major groups; doxin-like and non-dioxin-like PCBs. PCDFs and PCDDs are wide groups of doxin-like PCBs (15). Non-dioxin-like PCBs having 209 isomers are different in the number and position of chlorine atoms (1-10 atom) and 130 types of them are in use in commercial products but only 20 isomers of PCBs exist practically, among them, the numbers of PCB28, PCB52, PCB101, PCB118, PCB138, PCB153, and PCB180 (IUPAC) are recognized as indicator in environmental contamination. It should be emphasized that Iran is a member of Stockholm convention and is obligated to obliterate Polychlorinated biphenyls until the year 2025 (16). On the other hand, FAO and WHO institutions have setup strict regulations for using POPs. Although the usage of DDTs, HCHs, and Cyclodienes insecticides has been banned since 1980s, DDTs are still being used in low amount in some countries like china (17, 18). For this reason determination of POPs and monitoring of their environmental existence are necessary for risk assessment purposes (19).

Due to extremely low levels of POPs in food matrices, determination of OCPs and PCBs in food requires the application of effective extraction and sample purification techniques followed by a strong chromatographic and identification along with quantitation techniques (20). The analytical procedure of determination of OCPs and PCBs should be different and it consists of three steps: extraction, clean up, and chromatographic detection. Several techniques of determination of POPs are reported. The most common procedure for extraction of these organic compounds in a wide variety of matrices such as sediments, animal, and plant tissues is Soxhlet extraction (21-25). Soxhlet and matrix solid-phase dispersion (MSPD) (26, 27) are the traditional methods of extracting POPs from environmental samples. These procedures are carried out with large amounts of highly purified organic solvents and require long time (8-15h) for completing extraction. Microwave-assisted extraction (MAE) (28), pressurized liquid extraction (PLE) (29), accelerated solvent extraction (ASE) and supercritical fluid extraction (SFE) (30-36) are other extraction methods which are used for POPs. These techniques are suitable for dirty matrices and they can reduce the total time of extraction to about 2 h but the high cost of the equipment which are required for them, is disadvantage of these techniques. Membrane-assisted solvent extraction (MASE) is a technique that is discovered by Gerestel and it is very suitable for extracting organic pollutants like POPs in different aqueous samples (37-41). Low solvent consumption and minimal time requirements are the characteristic of this method.

Some other methods like QuEChERS have been extended in the last decade (42-44). This analytical method covers a very wide scope of polar, nonpolar, and semi polar analytes in various food matrices. Single phase extraction and the need to use various chemicals in sample preparation are advantage and disadvantage of this method respectively.

Liquid-liquid extraction (LLE) via solid-phase extraction (SPE) on silica gel or Florisil is still the classical method for separating and lead to decrease in fatty acids, pigments and other interferences from organic compounds in lipid-rich samples.

Gas chromatography coupled to tandem mass spectrometry (GC-MS/MS) techniques is one of the powerful instruments for monitoring of POPs residues in foods.

The present study aims to evaluate the occurrence of the most important persistent organic pollutants such as organochlorine pesticides and polychlorinated biphenyl congeners in trout by using highly sensitive and selective GC-MS/MS multi-residue method.

The extract undergoes a clean-up step using silica chromatography. The cleaned extracts are analyzed by means of GC-MS/MS and quantification takes place on the basis of the added internal standards.

## Experimental

*Sample collection*

Fish samples were collected from different regions of local markets in Tehran, Iran during 2 years (November 2015-December 2017). The samples were immediately transported to the laboratory and packed in clean plastic containers, then frozen at -20°C until use. Prior to the analysis, the samples were allowed to thaw and after removing skin, bones, head and tail, the fish filets were homogenized by kitchen grinder and stored at glass bottle until analysis.

*Chemicals (Reagents and Materials)*

The mixture of seven indicator PCBs (IUPAC nos: 28,52,101,118,138,153 and 180) and their isotope labelled standards in isooctane were purchased from Wellington Laboratories (Canada); Eleven OCPs standards (HCB, Dieldrin, Methoxychlor, α-, ϒ-Chlordane, α-, β-Endosulfan and *o,p’*-DDE, *p,p’*-DDE, *o,p’*-DDT and *p,p’*-DDT (the sum expressed as DDTs)) as individual ampoules in different solvents according to analysis paper, *p,p’*-DDE-D8, *p,p’*-DDT-D8 as IS for OCPs, were obtained from Chiron (Campro Scientific, Germany). All solvents used for the analyses including cyclohexane, dichloromethane, isooctane, *n*-hexane, and acetone were of SupraSolvgrade (Merck-Germany). Sodium chloride, anhydrous magnesium sulfate and silica gel were supplied by Merck (Germany) in analytical grade. Water was purified by a Milli-Q purification system with a minimal resistance of 18.2 MΩ/cm.

*Preparation of standards*

Individual stock solutions of OCPs were prepared in different solvents (Methoxychlor and α-, ϒ-Chlordane in cyclohexane, α-, β-Endosulfan in isooctane and HCB, Dieldrin and ΣDDTs in dichloromethane) at a concentration of 10 µg/mL (0.01 mg/mL). Stock solutions of 7 PCB congeners were made at 1 µg/mL in isooctane. For the seven ^13^C_12_-PCBs a solution of 0.5 µg/mL and for OCPs IS individual stock solutions at 10 µg/mL, were prepared in isooctane. A mixed intermediate stock solution containing 0.5 µg/mL of OCPs and 0.1 µg/mL of PCBs (in isooctane) was used for calibration curve was made. Calibration standard solutions in isooctane were prepared at the following concentrations: 0.1, 0.25, 0.5, 1, 2, 5, 10, 15, 20 and 30 ng/mL for PCBs and 5 times of these concentrations for OCPs. Concentration of internal standards was 25 ng/mL.All standard solutions were prepared in amber volumetric flasks and stored at 4 °C protected from light.

*Extraction and clean-up*

One gram of grinded fillet was added to falcon tube followed by the addition of internal standards (50 µl of the ISTD solution (500 ng/mL) of PCBs and 25 µL of the ISTD solution (1000 ng/mL) of OCPs) and allowed to equilibrate for at least 2 mins. 5mL distilled water was then added and shaken (Labinco– Netherlands) thoroughly until a slurry was obtained. 10mL *n*-hexane/acetone 1:1 (v/v) was added and vortex mixed for 1 min. Then 4 g MgSO4 and 2 g NaCl were added and mixed immediately for 1 min. The resulting mixure was centrifuged at 3600 rpm (Hettich Zentrifugen-ROTINA 380R – Germany) at 4 °C. One mL of the supernatant was transferred in Turbovap glass tube (Turbovap П Biotage 4001251- Sweden) and evaporated to dryness and the remaining residues was dissolved in 1 ml *n*-hexane and subjected to the sample clean-up process as follow:

Clean-up of the crude extract was accomplished by solid-phase extraction (SPE). A glass column was prepared using 1 g of deactivated silica. The silica was deactivated by washing with DCM 3 times and placing on adjustable oven (Memmert– Germany) at 250 °C for 24 h. The column was conditioned using 10 mL *n*-hexane, 5 mL *n*-hexane/DCM 1:1 (v/v) and 5 mL *n*-hexane respectively. The sample was transferred quantitatively on the column and rinsed 2 times with 1 mL of *n*-hexane, then eluted with 10 mL *n*-hexane/dichloromethane 1:1 (v/v) and the eluted solution was collected in a Turbovap tube. The eluate was concentrated to 0.5 mL by Turbovap instrument and the glass tube was rinsed with 2 mL isooctane. After being homogenized by using vortex, the eluate was concentrated to 0.5 Ml again and transferred to GC-MS vial in a 200 µL insert.

*GC-MS analysis*

The detection and qualification of all POPs in this study were performed by using an Agilent 7890A GC coupled to an Agilent 7000 triple-quadrupole mass spectrometer with electron impact ionization (EI, 70 ev) adjusted for multiple reaction monitoring mode (MRM) at a different collision energy for each analyte. The ion source and MS transfer line temperatures were set at 230 and 300 °C, respectively. Gas chromatography system was equipped with 7693 autosampler (Agilent technologies). The eighteen POPs were separated by using HP-5MS capillary column (30m×0.25mm id×0.25 µm film thickness). Inlet was set in splitless mode and temperature of injection port and volume of injection were 300 °C and 1µL, respectively. 

**Table 1:** Qualifier and quantifier ions used in the multiple reaction monitoring (MRM) of OCPs, PCBs and there is, analyzed by GC-MS/MS

**Component**	**Precursor ions**	**Product ions (Quantifier/Qualifier)**	**Collision energy ** **(ev)** **a**
HCB	285	249/214	16/15
*α*-Chlordane	410	266/301	28/15
γ -Chlordane	410	266/301	28/15
Dieldrin	380	193/228	35/24
o,p'-DDE	318	176/248	28/15
p,p'-DDE	318	176/248	28/15
o,p'-DDT	354	165/199	30/15
p,p'-DDT	354	165/199	30/15
*α*-Endosulfan	406	206/159	15/10
ß-Endosulfan	406	206/160	13/10
Methoxychlor	345	141/169	35/35
PCB 28	256/258*	186	30
PCB 52	290/292*	220	30
PCB 101	326/328*	256	35
PCB 118	326/328*	256	35
PCB 153	360/362*	290	30
PCB 138	360/362*	290	30
PCB 180	394/396*	324	35
p,p'-DDE-D8	326	184/256	28/15
p,p'-DDT-D8	362	173/207	30/15
13C-PCB 28	268/270*	198	30
13C—PCB 52	302/304*	232	30
13C-PCB 101	338/340*	268	35
13C-PCB 118	338/340*	268	35
13C-PCB 153	372/374*	302	30
13C-PCB 138	372/374*	302	30
13C-PCB 180	406/408*	336	35

*The second ion transition was used as qualifier transition.

a Electron volt

**Table 2:** Performance characteristics of the method for analysis of POPs (OCPs and PCBs) in fish using LLE procedure and MRM method of GC-MS/MS in blank trout at five spiking levels (n = 3)

**Analyte**	RT*(min)	**LOD ** **a ** **(ng/g)**	**LOQ ** **b ** **(ng/g)**	**Recovery ± RSD (%) ** **c**	**Uncertainty (%)**
HCB	15.6	1.6	5	87.0 ± 3.4	6.78
*α*-Chlordane	19.5	3.3	10	85.2 ± 9.3	18.58
γ -Chlordane	19.3	1.6	5	108.5 ± 13.8	27.64
Dieldrin	19.9	3.3	10	100.8 ± 14.3	28.60
o,p'-DDE	19.3	1.6	5	103.9 ± 13.9	27.82
p,p'-DDE	19.8	1.6	5	111.8 ± 5.5	11.04
o,p'-DDT	20.5	3.3	10	95.8 ± 1.4	2.77
p,p'-DDT	21.0	3.3	10	109.7 ± 13.5	27.01
*α*-Endosulfan	19.5	8.3	25	73.3 ± 17.9	25.70
ß-Endosulfan	20.4	8.3	25	85.2 ± 5.3	10.55
Methoxychlor	21.8	3.3	10	83.2 ± 14.5	29.08
PCB 28	17.1	0.6	2	89.0 ± 4.4	8.82
PCB 52	17.7	0.6	2	84.4 ± 2.9	5.74
PCB 101	19.3	0.6	2	80.5 ± 8.1	16.14
PCB 118	20.3	0.6	2	87.3 ± 7.3	14.67
PCB 138	20.7	0.6	2	82.0 ± 14.7	29.47
PCB 153	21.1	0.6	2	95.1 ± 8.5	17.08
PCB 180	22.1	1.6	5	76.0 ± 15.4	30.88

*Retention time (minute)

a LOQ, Limit of quantification for a S/N = 10

b LOD, Limit of detection for a S/N = 3

c According to wet weight

**Table 3:** Occurrence of OCPs and PCBs in fish samples from Tehran markets (n = 15)

**Analyte**	**Incidence (%)**	**Mean**	**Median**	**Maximum**	**Maximum Residue Limits (MRLs) (ng/g) wet weight**
HCB	Nd*	< LOQ	< LOQ	-	200
*α*-Chlordane	Nd*	< LOQ	< LOQ	-	
					50 (Sum of cis- and trans-isomers)
γ -Chlordane	Nd*	< LOQ	< LOQ	-	
Dieldrin	Nd*	-	-	-	200 (Singly or combined with Aldrin)
o,p'-DDE	Nd*	< LOQ	< LOQ	-	
p,p'-DDE	20	12.83	15	17.9	1000 (Sum of o,p-DDT, p,p-DDT, p,p-DDE and p,p-TDE (DDD))
o,p'-DDT	Nd*	< LOQ	< LOQ	-	
p,p'-DDT	6.66	10.2	10.2	10.2	
*α*-Endosulfan	Nd*	-	-	-	50 (Sum of alpha and beta endosulfan and endosulfan sulphate)
ß-Endosulfan	Nd*	-	-	-	
Methoxychlor	Nd*	-	-	-	10
PCB 28	Nd*	< LOQ	< LOQ	-	
PCB 52	Nd*	-	-	-	
PCB 101	Nd*	-	-	-	
PCB 118	Nd*	-	-	-	75 (Sum of PCBs)
PCB 138	Nd*	-	-	-	
PCB 153	Nd*	-	-	-	
PCB 180	Nd*	-	-	-	

* Not detected

**Table 4.** Comparison of LLE method used in the present study with other reported methods for determination of 18 POPs in fish samples

**Authors**	**Extraction method**	**Detection system**	**LOD (ng/g)**	**LOQ (ng/g)**	**ISTD**	**Recovery (%)**	**RSD (%)**	**V**_L_^a^ **(mL)**	**Extraction solvents**	**Ref.**
Cocco *et al.*	PLE	GC-MS	20 ^b^	40 ^b^	PCB 209	74-94	11-19 ^c^	90	n-Hexane	
Munshi *et al.*	MSPD	GC-ECD	0.01-1	-	-	90-120	4.1-6	25	n-Hexane	
Schmid *et al.*	GPC	GC-MS	-	-	^13^C_12_-PCBs, ^13^C_12-_OCPs	-	-		-	
Suchan *et al.*	PLE (Compare Soxhlet) -GPC	GC-ECD	-	-	-	90-120	3-14	40-170	H/D,H/A 1:1,4:1	
Voorspoels *et al.*	Soxhlet	GC-ECD GC-MS	-	0.01	PCB 46,143	-	< 30	80	H/A 3:1	
Covaci *et al.*	Soxhlet	GC-ECD	-	0.1-0.4	PCB 46,143	86-120	< 30	75	H/A,H/D 3:1,1:1	
Drabova *et al.*	PLE-GPC	GC-MS (TOF)	-	0.1-0.5	^13^C_12-_PCB 77	74-118	4-12	20-40	H/D 1:1	
Yang *et al.*	ASE	GC-ECD	-	-	^13^C_12_-PCBs	-	-	70	H/D 1:1	
Davodi *et al.*	Soxhlet	GC-ECD	-	0.1-0.3 ^b^	^-^	90-110	<10	100	H/A 3:1	
Lui *et al.*	GPC	GC-HRMS	-	-		94-122	0.7-22	150	H/D 1:1	
Method described in this study	LLE	GC-MS/MS	0.6-8.3	2-25	^13^C_12_-PCBs, DDE-D4 DDT-D4	73-112	1.4-18 (or < 20)	10	H/A,H/D ^d^ 1:1,1:1	This paper

^a^ Extraction volume

^b ^lipid weight

^c ^SD

^d ^n-Hexane/Acetone, n-Hexane/Dichloromethane

**Figure 1:**
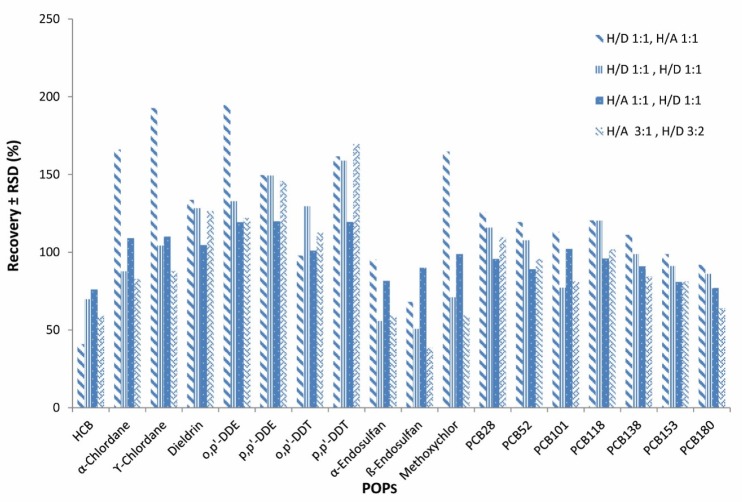
Effect of various extractants and the extraction solvent volume (n = 4) on the average recoveries and repeatability (RSD) of the analytical method for OCPs and PCBs

H/D: n-Hexane-Dichloromethane, H/A: n-Hexane-Acetone.

**Figure 2:**
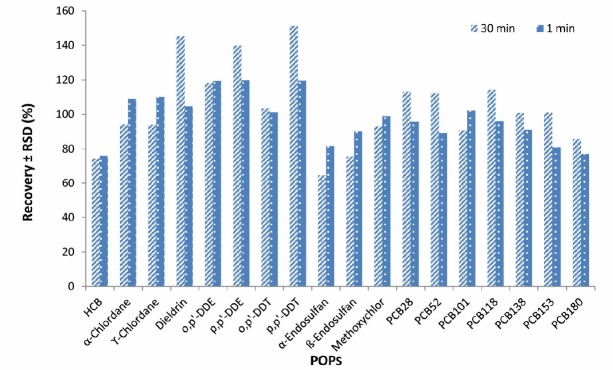
Effect of extraction time (n = 2) on the average recoveries and repeatability of the analytical method used for analysis of PCBs and OCPs using GC-MS/MS

**Figure 3:**
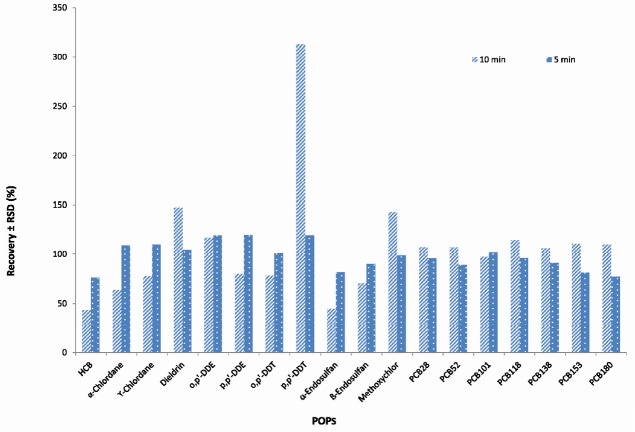
Effect of centrifugation time (n = 3) on the average recoveries and repeatability of POPs analyzed in fish samples using GC-MS/MS

**Figure 4:**
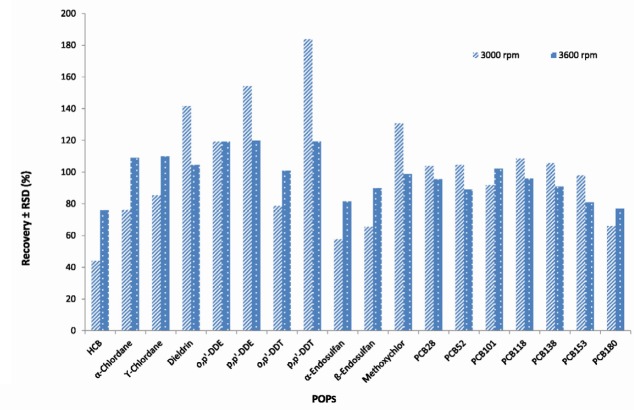
Effect of centrifugation rate (n = 3) on the average recoveries and repeatability of OCPs and PCBs analyzed in fish samples using GC-MS/MS

**Figure 5:**
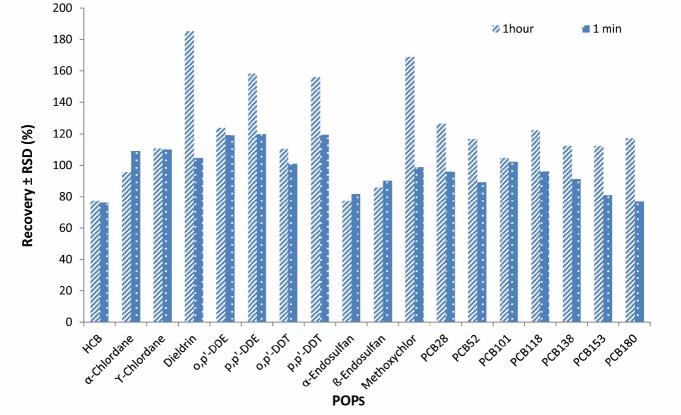
Effect of elapsed time after adding the salts (n = 2) on the average recoveries and RSDs of OCPs and PCBs analyzed in fish samples using GC-MS/MS

**Figure 6:**
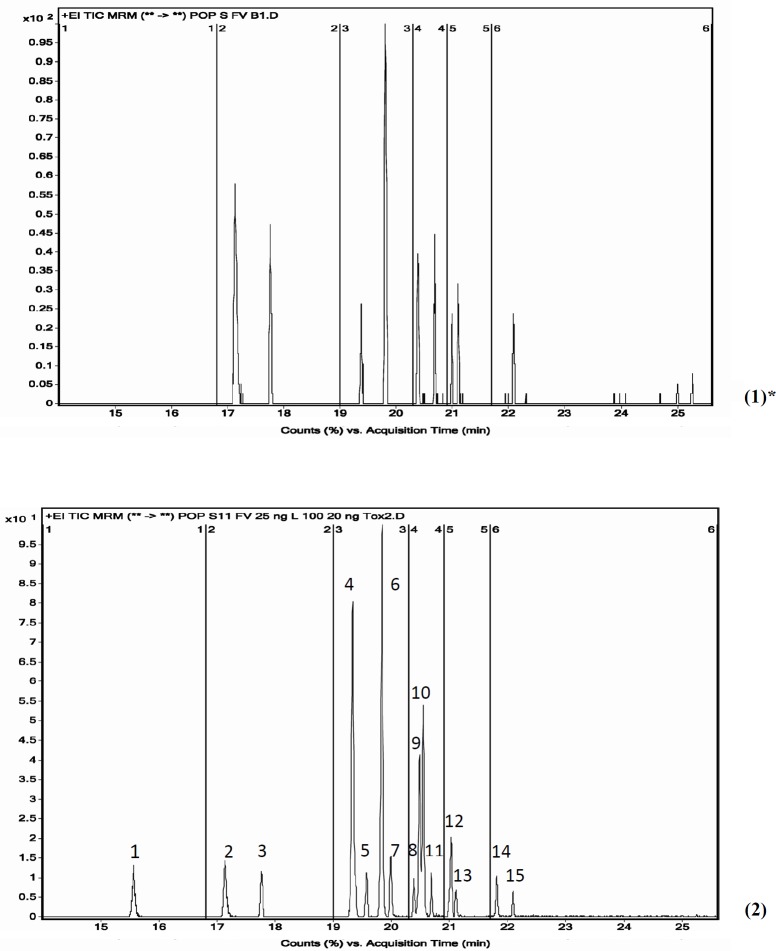
GC-MS/MS chromatograms of a blank fish sample (1) and a fish sample spiked with 11 OCPs at 100 ng/gr and 7 PCBs at 20 ng/gr (2)

1: HCB, 2: PCB 28, 3: PCB 52, 4: PCB 101, ϒ-Chlordane, *o,p’*-DDE, 5: α -Chlordane, α -Endosulfan, 6: *p,p’*-DDE, 7: Dieldrin, 8: PCB

118, 9: β-Endosulfan, 10: *o,p’*-DDT, 11: PCB 138, 12: *p,p’*-DDT, 13: PCB 153, 14: Methoxychlor, 15: PCB 180.

*Blank chromatogram contains ISIDs (13C -PCB (28-52-101-118-138-153-180), *p,p’*-DDE-D8 and *p,p’*-DDT-D8).

The carrier gas with high purity (99.999%) was helium at constant pressure in the pressure program as follows: 7psi for 1 min, raised at a rate of 10 psi min^-1 ^to 1.5 psi, raised at a rate of 0.4 psi min^-1 ^to 9.6 psi and then raised at a rate of 1 psi min^-1 ^to 11.2 psi and held for 1min. The temperature program of HP-5MS column was set to initial temperature of 60 °C for 2 min, raised at a rate of 20 °C min^-1 ^to 120 °C, raised at a rate of 10 °C min^-1 ^to 260 °C and then raised at a rate of 25 °C min^-1 ^to 300 °C and held for 5 min. Helium and nitrogen were used as the quench and collision gas at constant flow (1 ml min^-1^). The qualifier and quantifier ions used in MRM mode are presented in Table 1.

*Method validation*

Validation of the method was confirmed using the analytical parameters as recovery, limit of detection (LOD), limit of quantification (LOQ), linearity, accuracy, and precision. Calibration curves were obtained over the concentration range of 0.1-30 ng/mL and 0.5-150 ng/mL for PCBs and OCPs, respectively. In the present study the recoveries were determined for five concentration levels of 1, 2, 5, 10, and 20 ng/g for PCBs and 5-100 ng/g for OCPs in five spiked blank trout in triplicates and three consecutive days. ^13^C_12_-PCB 118 was used as internal standard for all OCPs. For DDTs; however, their stable isotopes labeled derivatives were used as internal standards for calculating recovery. Relative standard deviations (RSDs) were calculated using the results of recoveries in 3 different. LODs and LOQs were attained based on the signal-to-noise ratios of 3:1 and 10:1, respectively.

*Method optimization*

*Extraction Solvent*

For optimization of the method to achieve the best selective solvent, several mixtures of solvents as 1 for preparation: *n*-hexane/DCM 1:1 (v/v) and for clean-up: *n*-hexane/acetone 1:1 (v/v), 2 for preparation: *n*-hexane/DCM 1:1 (v/v) and for clean-up: *n*-hexane/DCM 1:1 (v/v), 3 for preparation: *n*-hexane/acetone 1:1 (v/v) and for clean-up: *n*-hexane/DCM 1:1 (v/v) and 4 for preparation: *n*-hexane/acetone 3:1 (v/v) and for clean-up: *n*-hexane/DCM 3:2 (v/v) (42), were checked and number 3 was selected as the preferred solvent mixture (Figure 1).

The optimum volume of extraction solvent was determined as 10 mL and decreasing the volume to 8 and 5 mL did not give acceptable results.

*Extraction time*

Increasing the time of vortex shaking by vortex from 1 to 30 min did not have a significant effect on extraction efficiency and therefore, 1 min was selected as an extraction time (Figure 2).

*Centrifugation Time and Rate*

The effect of centrifugation time time (1, 5 and 10 min) and rate (1500, 3000 and 3600 rpm) on extracting and rate on extracting efficiency were also examined using 2, 5, 10 ng/g concentration levels for PCBs and 10, 25, 50 ng/g for OCPs. The best results were obtained using centrifugation of the extract at 3600 rpm and 5 minutes (There was no separation in 1500 rpm and 1 min) (Figures 3, 4).

*Influence of the elapsed time after adding the salts*

Three concentration levels of POPs (2, 5, 10 ng/g PCBs and 10, 25, 50 ng/g OCPs) were examined for checking the effect of increasing the elapsed time after addition of the salt. The results showed no significant improvement in extraction efficiency when the elapsed time was increased from 1min to 1 h. The recovery results are shown in Figure 5.

## Results

*Method validation*

Calibration standards at concentration levels of 0.1-30 ng/mL for PCBs and 0.5-150 ng/mL for OCPs were made by addition of internal standards at a concentration of 25 ng/mL (IS POPs). 

The correlation coefficients were between 0.990 and 0.999 which confirmed a linear relationship between the concentration and peak ratios.

Precision and accuracy were measured by RSD% and recoveries at five concentration levels (5, 10, 25, 50, and 100 ng/g OCPs, 1, 2, 5, 10, and 20 ng/g PCBs), respectively. The results were within the acceptable range of RSD, recommended by EU Commission (59-63).

LODs and LOQs were calculated by measurement of signal-to-noise and the values were between 0.6-8.3 µg/kg for LODs and 2-25 µg/kg for LOQs.

The extraction recovery of target POPs at 5 QC levels were determined by comparing ratio values (area under the curve of analyte relative to the area under the curve of internal standards) in the spiked sample. The samples were all run in triplicate (n = 3) within a day and three different days. 

The validation parameters for analysis of OCPs and PCBs in fish samples using LLE procedure and GC-MS/MS method are summarized in Table 2.

*Application of the optimized method for the analysis of unknown fish sample*

To demonstrate the applicability and the suitability of the developed method, the real fish samples were analyzed for the existence of POPs. For determination of values of POPs in 15 samples of trout, 1 g of fillet was prepared by LLE extraction method and analyzed by the validated method. The results indicate that POPs were not detected in 2 fish samples and for the other samples, the amounts of the POPs were below Maximum Residue Limits (MRLs).

The results of occurrence of OCPs and PCBs in fish are shown in Table 3.

## Discussion

The method of extraction and purification used in this paper is one of the easiest and most accessible methods of analysis. Limited use of solvents, short analysis time, and no need for special equipment and expensive devices are some of the benefits of the method used in this article. This method is divided into two parts: extraction processes and clean up by LLE and SPE (silica column), respectively. In the first part, using the mixture of hexane-acetone solvent and MgSO4 and NaCl salts, extraction of the desired organic pesticides and their salting out from the water in the sample, is implemented. In the cleanup part classical liquid adsorption chromatography involving silica gel is used to remove polar organic materials, lipids, water excess, and all other interactions. The extracts thus obtained, are analyzed by gas chromatography coupled to mass spectrometry.

The other extraction methods like Soxhlet, GPC, MSPD, PLE, and ultrasonic extraction (USE), have been used for detection of POPs in fish and fishery products. Soxhlet method is a traditional procedure for analysis of POPs, whose one of problem is the high solvent consumption and long extraction time. Table 4 shows the comparison of the current method with other published methods used for analysis of OCPs and PCBs in fish samples.

As seen in Table 4, extraction time and volume of solvent consumed in our method were in the lowest amount, therefore it could be considered as an attempt to make the extraction method greener. Also, our method does not require any particular device and equipment for sample preparation, but some of the other methods listed in the Table 4, such as PLE, ASE, GPC, MSPD, and Soxhlet, require very specialized equipment (15, 27, 29, 45-51). The type of extraction solvents used and their percentages are very important. In the optimization method section, several types of solvents have been tested to select the best solvent mix which was finally determined to be number 3 (H/A 1:1 ,H/D 1:1). Figure 6 shows GC-MS/MS chromatogram of POPs in blank and spiked fish sample.

According to different methods in table 4, there are a few papers using multiple internal standards for analysis of PCBs and OCPs. In this study, 7 IS for PCBs and 2 IS for OCPs were used which provided a high level of accuracy for the analytical method.

The high frequency and high levels of contamination with DDTs, HCB, and PCB 28 could be due to the lipophilicity as well as chemical stability and thus environmental persistency of these toxins compared to the other ones (Table 3) (52, 53).

One of DDTs isomers is p,p’-DDE metabolite. A high percentage of total DDT reported, belongs to this metabolite. The reason is aerobic breakdown of p,p’-DDT metabolite by microorganisms. Moreover, p,p’-DDE metabolite is very persistencet in marine ecosystems and has high accumulation in fat tissues (54). It shall be noted that as the result of p,p’-DDT anaerobic breakdown, p,p’-DDD metabolite is also produced but its amount is less than p,p’-DDE (55).

This result is in conformity with the similar researches in other countries (56).

Hexachlorobenzene does not exist naturally in the environment. It is produced in the process of producing chemical materials such as solvents and as an additive in the formulation of some pesticide including pentachlorophenol. Improper waste disposal in rural area (such as garbage incineration) which results in HCB formation in the environment could be one of the factors which cause the high level of contamination of river water (6 µg/L) and thus the living fish in them (57, 58). 

As it is seen in table 3, α-Chlordane and ϒ-Chlordane toxins were only found once in the examined fishes. Considering the widespread use of chlordane in the past and its one-year life time in soil, the observed contamination in the present study could be due to the application of chlordane in the past.

Determination of the residues of persistent organic pollutants in trout collected in around Tehran indicates that the pollution is lower than maximum residue levels (MRLs) set by European Council Directives in fishes and aquamarines (59-63). It should be noted that the amount of POPs intake depends on the food dietary regimen as well as the daily consumption of trout by each individual. The results indicated that the method performance characteristics were in the acceptable range (56-63).

## Conclusion

In the present research, LLE method has been used as one of the easiest and least costly methods for analyzing PCBs and OCPs. In addition, using the MRM mass monitoring mode has provided a great selectivity for measuring persistent organic pollutants. On the other hand, the application of seven internal standards for polychlorinated biphenyls (^13^C_12_-PCB) and two internal standards for DDTs (*p,p’*-DDE-D8,* p,p’*-DDT-D8), have provided a high level of accuracy of the analyses.

The simple internal standard calibration method which has been employed in the present study is another advantage of the method compared to the more complicated matrix match and spiked calibration methods.

Quantitation analysis factors like recovery percentage, precision, and correlation coefficient were within the acceptable range of European Commission regulation (59-63).

Primary results indicated the contamination of fish samples with toxins specially DDE and DDT. Considering the fact that uses of DDTs are banned by agricultural authorities, the presence of these toxins in fish indicates the illegal use of these pesticides at least in some agricultural activities.

Due to the presence of contamination in trout, a more comprehensive survey of fish contamination in all parts of Iran seems to be necessary.
